# Decaying Logs Shape the Distribution of Bird‐Mediated Seed Rain in a Temperate Deciduous Forest

**DOI:** 10.1002/ece3.74087

**Published:** 2026-07-29

**Authors:** Przemysław Kurek

**Affiliations:** ^1^ Department of Plant Ecology and Environmental Protection Adam Mickiewicz University Poznań Poland

**Keywords:** distribution, logs, perching, seed dispersal, seed rain

## Abstract

Birds contribute to seed dispersal through endozoochory, often creating spatially aggregated patterns of seed deposition around perching sites. While this process has been extensively studied in open habitats, the role of deadwood in structuring bird‐mediated seed rain within forest ecosystems remains poorly understood. This study investigated whether decaying logs function as important perching sites which influence the distribution of bird droppings and associated seed rain in a temperate deciduous forest. Bird droppings were collected during three seasons in 2021–2023 in an oak‐hornbeam forest, using seed traps placed either beneath decaying logs or at randomly selected forest‐floor locations. A total of 524 droppings were recorded. The abundance of droppings was significantly higher beneath logs than in random plots throughout the study period, with 73.7% of all droppings found in log‐associated plots. This created a strongly aggregated pattern of seed deposition, as 80.6% of all recovered seeds occurred beneath logs. Overall seed dispersal by birds was low. Only 4.6% of droppings contained seeds, and mean seed rain reached 0.39 seeds × m^−2^ × season^−1^. These findings suggest that although bird‐mediated seed input is limited, its distribution is strongly shaped by deadwood availability. Decaying logs therefore represent an overlooked structural component linking birds and fleshy‐fruited plants.

## Graphical abstract

Sites used by birds for perching and roosting function simultaneously as defecation points, concentrating diaspores transported via endozoochory. In contrast, the role of perching structures in shaping bird‐mediated seed rain within forest ecosystems remains poorly understood. Logs may act as exposed microhabitats attractive to frugivorous birds as excellent perching sites and potentially serve as focal points of seed deposition.

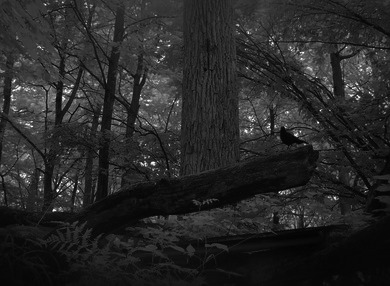

## 1 Introduction

Sites used by birds for perching and roosting are also defecation points, concentrating diaspores transported via endozoochory (McDonnell and Stiles 1983). In semi‐natural habitats, especially on landscapes dominated by cropland, sites such as woodlots, solitary trees, and artificial structures (e.g., pylons) become focal points of seed deposition (Herrera and García 2009). As a consequence, many fleshy‐fruited plant species, both native and alien, whose diaspores were transported with bird droppings, regenerate under those various natural and artificial perches (Bonilla and Pringle 2015). This process, often described as nucleation (Pausas et al. 2006), is a well‐established driver of secondary succession in open habitats (McDonnell and Stiles 1983; Kurek et al. 2015).

The role of perching structures in shaping bird‐mediated seed rain within forest ecosystems is still poorly understood. Canopy gaps have been considered the main driver maintaining the bird‐generated seed shadow (Thompson and Willson 1978), but down woody material has received little attention. Coarse woody debris as seed and seedling nucleation points has only been analyzed in relation to acorn caching by corvids (synzoochorous dispersal) (Castro et al. 2012). Intensification of forestry and the resulting homogenization of forest landscapes have led to the reduction and elimination of many structural elements, such as logs and standing dead trees, with negative effects on forest biodiversity (Kapusta et al. 2020). Logs may provide exposed microhabitats attractive to frugivorous birds as perching sites and thus potentially may serve as focal points of seed deposition through droppings or regurgitation (see also: Kurek et al. 2026). They can play a role similar to single trees in mixed habitats visited by birds leaving their droppings (Herrera and García 2009). Birds visiting and perching on logs increase the probability of seed deposition and subsequent seedling establishment of fleshy‐fruited plant species. The aim of this study was to evaluate whether decaying logs function as significant perching sites influencing the spatial distribution of bird droppings and the associated seed rain in a temperate deciduous forest. Two hypotheses were tested: (1) logs are important places for birds to perch, increasing the local abundance of their droppings, and (2) logs modify the spatial pattern of bird‐mediated seed dispersal (seed rain) in the studied forest ecosystem.

## 2 Methods

### 2.1 Study Area

The research was carried out in a natural and strictly protected old oak‐hornbeam forest located in the Dębina Reserve 50 km north of Poznań, Poland (52.80° N, 17.14° E) on a flat area at ca. 80 MASL, with 8.0°C–8.5°C mean annual temperature, 500–600 mm annual precipitation and a vegetation period averaging 210–220 days (Balcerkiewicz et al. 1977). The soils are developed from sorted fluvioglacial deposits differentiated in texture both horizontally and vertically. These are layered sand and gravel deposits with pebbles in a layer 2–10 m thick. They consist of sands or slightly loamy sands, often with admixture of gravel, accumulated in an ice‐marginal valley on impermeable and lime‐rich tills (Wojterski 1976).

The reserve's protected status dates back to 1933 when its core area was established. In 1957 the entire area was placed under protection; no forest management is allowed there now—only natural processes are the main drivers of the forest structure. The discussed oak‐hornbeam *Galio‐Carpinetum* forest in mesic and eutrophic habitats is dominated by ca. 300‐year‐old pedunculate oak *Quercus robur* creating the upper canopy layer. The estimated volume of down woody material (hereafter: DWM) there is 251.6 m^3^/ha (Kurek et al. 2026), much higher than the average reported for managed forests, which has been reported to range from 0.0–1.0 m^3^/ha (Bobiec 2002) to 4.9 m^3^/ha (WISL 2022). DWM covers approximately 2.4% of the forest floor area (Kurek et al. 2026). The distinguishing feature of this forest complex is the great diversity of admixture tree species, reaching up to 20 taxa (author's unpublished data), some of which reach about 180 years in age (BDL 2024). The most common are hornbeam *Carpinus betulus* , lime *Tilia cordata* , beech *Fagus sylvatica* , sycamore *Acer pseudoplatanus* , and field maple *A. campestre* . There are also patchily distributed moist habitats at lower elevations with European white elm *Ulmus laevis*, ash *Fraxinus excelsior* and also black alder *Alnus glutinosa* (Wojterski 1976). Due to low light access to the forest floor, typical for this type of deciduous forest in temperate Europe, shrub (mostly *Corylus avellana* ) and younger tree generation is unevenly and patchily distributed, mostly limited to canopy gaps and forest edges.

*Sambucus nigra* and *Prunus padus* are the most common woody species of the group of fleshy‐fruited plants that occur in the research area. The remaining fleshy‐fruited shrubs and trees, rare in the study area, are *Prunus serotina* (invasive alien), *Ribes uva‐crispa* , *Sorbus aucuparia* , *Rhamnus cathartica* , *Euonymus europaeus* , *Crataegus monogyna* , and *Rubus idaeus* . In the herb layer, the most common fleshy‐fruited species are *Polygonum multiflorum* , *Paris quadrifolia*, and *Convallaria majalis* . Some cultivated trees ( *Morus alba* , *Prunus domestica* , *P. cerasifera* , *Malus domestica* , *Pyrus* sp.) show a distribution associated with the settlements, orchards, and roadsides in the farmland surrounding the reserve mainly to the west and northwest.

### 2.2 Dropping Collection and Analysis

Bird droppings were collected in 2021–2023 during 22 surveys using 64 seed/dropping traps. Each trap consisted of a tray (40 × 120 cm, 0.48 m^2^) covered with wire mesh (mesh size 0.8 cm) to prevent postdispersal seed removal (González‐Varo et al. 2021). Of these, 32 seed traps were located beneath DWM (Appendix S1), including logs and thick branches with diameter > 10 cm at the thinner end (log plots). Potential sampling units were identified during field surveys by systematically searching the study area and locating all suitable pieces of woody debris that met the size criterion. From this pool, logs were selected to ensure a broad spatial distribution across the study site and to avoid clustering of sampling points. Only woody debris that was in stable contact with the ground and clearly distinguishable from surrounding structures was considered. Lying logs showing a very advanced degree of decomposition were excluded. In order to quantify the magnitude of bird‐mediated seed rain independent of fruiting individuals, the other 32 seed traps were placed at random forest‐floor locations not associated with fleshy‐fruited plants (random plots). The traps were checked every 15–20 days from June to November, the period covering the fruiting phenology of fleshy‐fruited herbs, shrubs, and trees. When heavy rains occurred, possibly destroying or rinsing out the droppings, the trays were checked again about a week later. During the 2023 field study, all logs were monitored with camera traps to determine whether frugivorous birds used them as perches. Monitoring was done from June to October to capture the presence of migratory bird species. The presence of perching birds was determined based on photographic records. Each photograph was treated as a separate observation. That analysis included only observations separated by a minimum interval of 15 min. Seeds were counted and visually identified using the author's reference collection and identification atlases (Cappers et al. 2006; Bojňanský and Fargašová 2007; Tomlik‐Wyremblewska et al. 2010). For simplicity, the term “seed” is used throughout to refer to different types of diaspores containing seeds, including drupes. All seed samples are stored at the Laboratory of Plant Ecology and Environmental Protection, Adam Mickiewicz University in Poznań, Poland.

### 2.3 Statistical Analysis

The response variable (number of feces) was analyzed using generalized linear mixed models (GLMMs). Plot type (log vs. random) and season were included as fixed effects, together with their interaction. Because repeated observations were collected from the same sampling units, tray identity was included as a random intercept to account for nonindependence of repeated measurements. The overdispersion ratio (> 1.5) indicated substantial extra‐Poisson variation (1408 observations, of which 63% were zeros). To account for overdispersion and the high proportion of zeros in the dataset, a zero‐inflated negative binomial model (family = nbinom2) was fitted using the “glmmTMB” package (Brooks et al. 2017). The negative binomial distribution allows the variance to exceed the mean, thereby accommodating overdispersion. Model fit was evaluated using AIC and inspection of model summaries. The significance of fixed effects in the conditional component was assessed using Wald *z*‐tests. Model coefficients were exponentiated to obtain incidence rate ratios (IRR), facilitating interpretation in terms of multiplicative changes in expected feces counts. Post hoc comparisons were calculated with the “emmeans” package (Lenth and Piaskowski 2025). Simple effects were analyzed to compare plot types within each season. Pairwise contrasts between factor levels were evaluated with Tukey's adjustment for multiple comparisons.

For categorical data the Chi square test (*χ* ^2^) of goodness of fit was used and based on the proportions between seed number for logs and for random plots. All analyses employed R software version 4.3.1 (R Core Team 2025). The Jaccard index was used to assess similarity between seeds of fleshy‐fruited plant aggregations from logs and random plots. This measure quantifies the degree of overlap between two assemblages by relating the number of shared species to the total number of species present in either set. The index ranges from 0 to 1, where 0 indicates no common elements and 1 denotes complete similarity between the compared sets.

## 3 Results and Discussion

**FIGURE 1:**
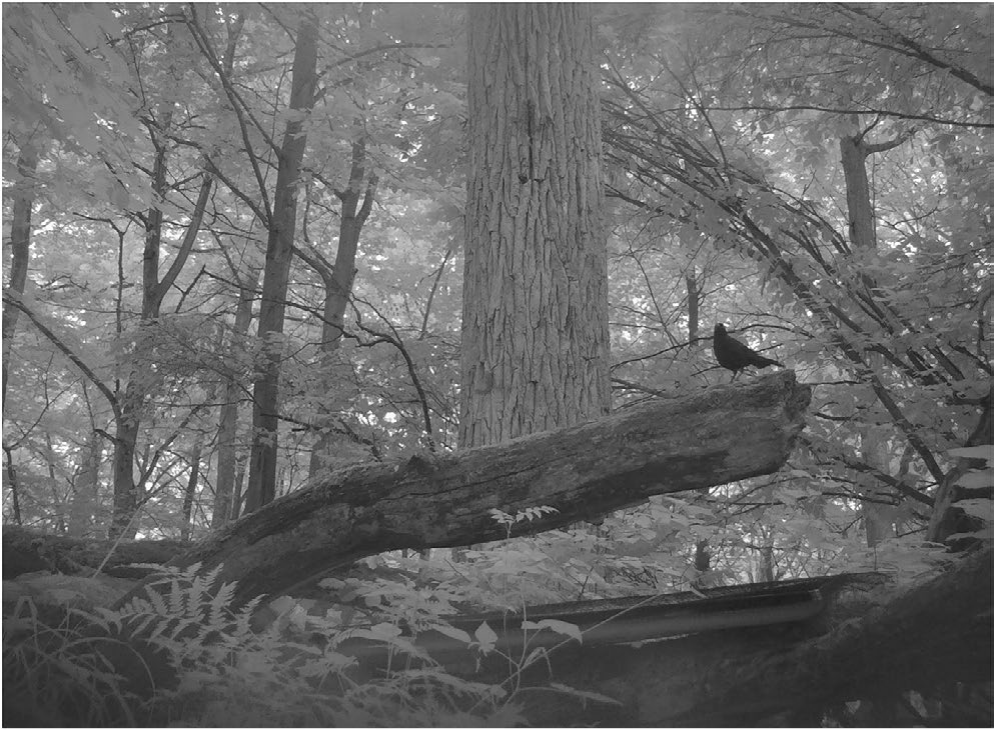
A blackbird *Turdus merula* perched on a log above a seed trap.

**FIGURE 2:**
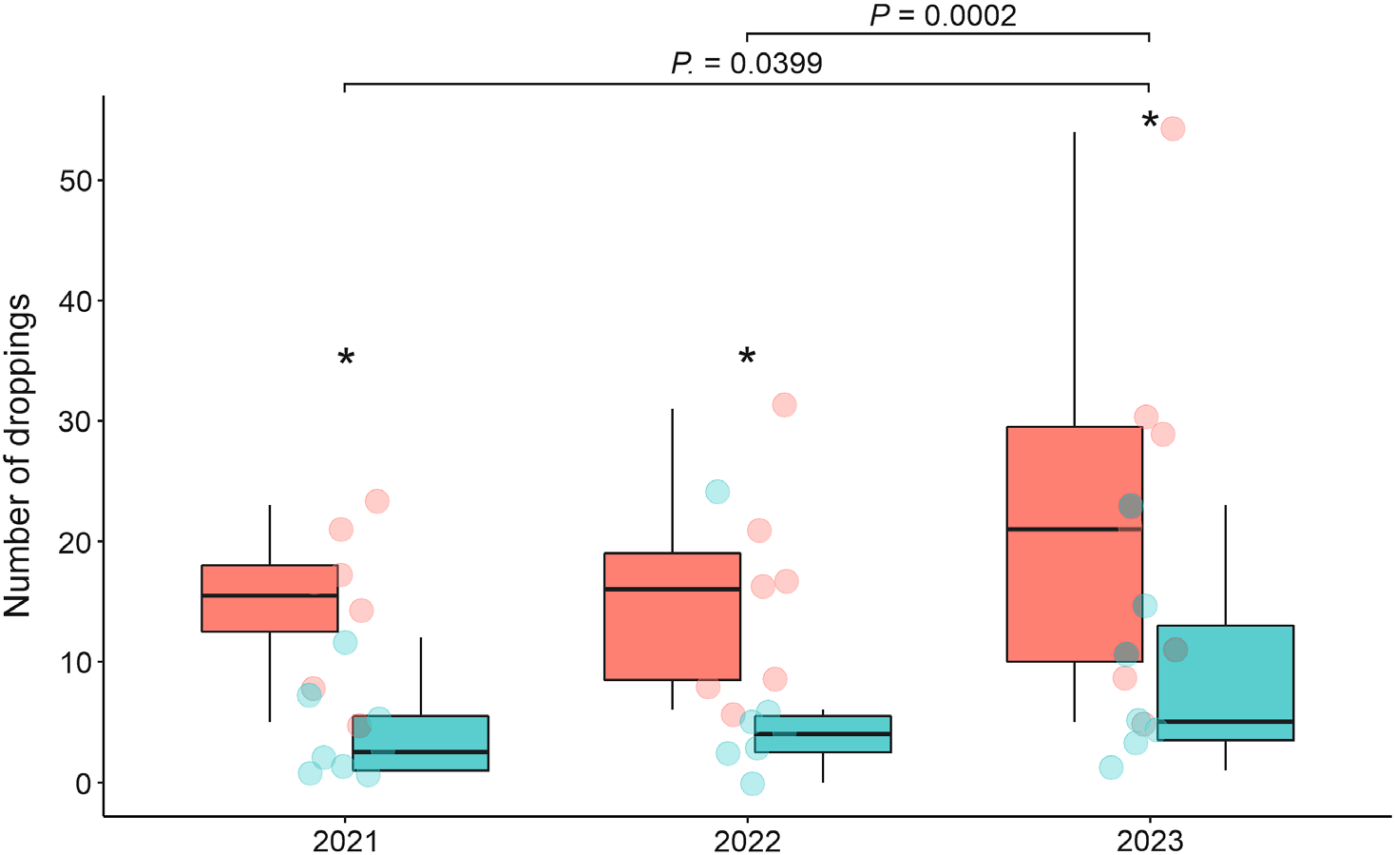
The number of bird droppings per survey differed significantly in season 2023 and between plot types (*p < 0.0001), with higher abundance in plots with logs (red) than in random plots (green) during 2021–2023.

**FIGURE 3:**
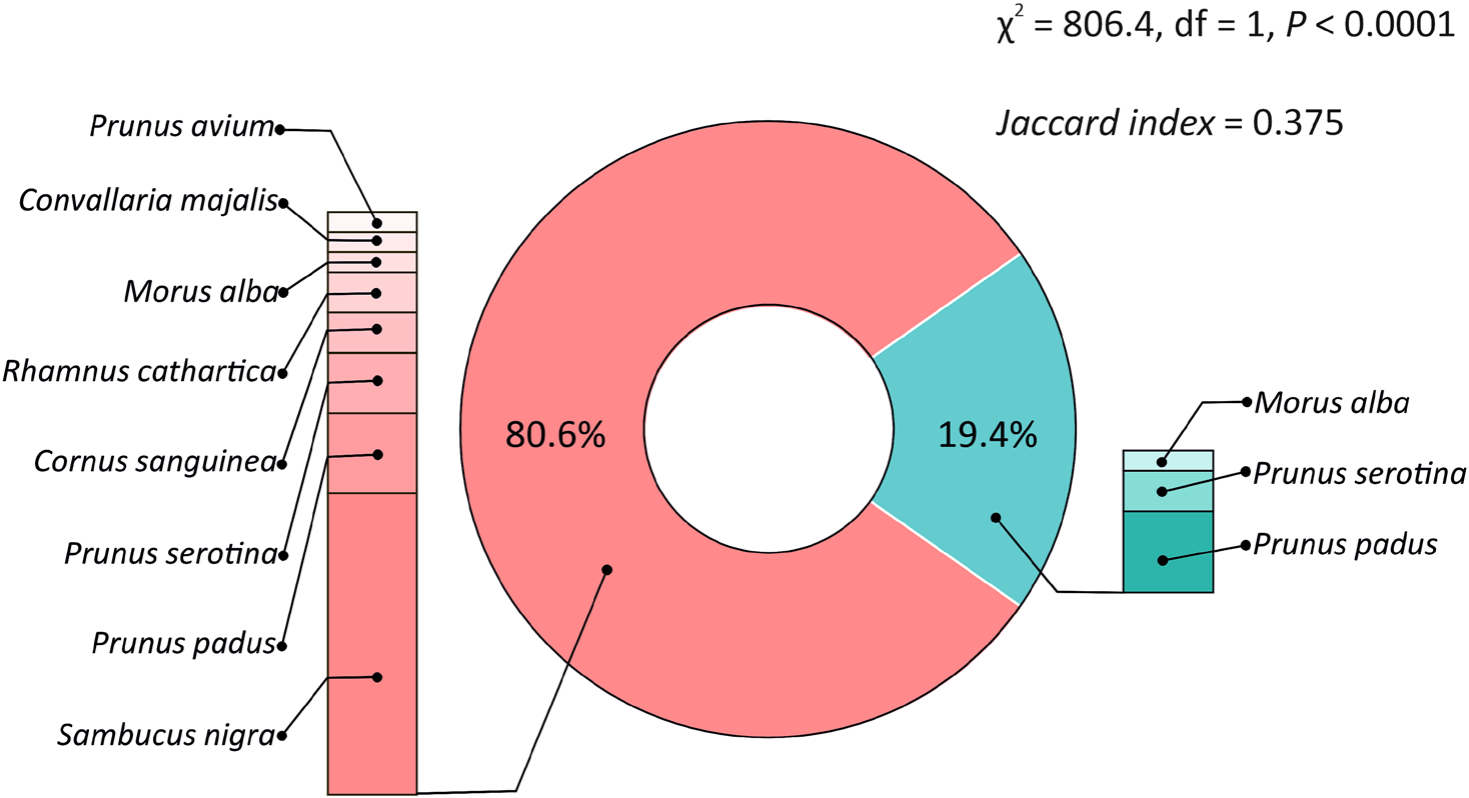
Proportion of seeds sampled from log plots (shades of red) and from random plots (shades of green), with clear predominance of seeds concentrated around logs; bars present species structure and similarity (Jaccard index) of seed composition from droppings.

Camera‐trap monitoring recorded 15 bird species using logs as perches (Figure 1, Appendix S2). Approximately 47% of the records (*N* = 334) consisted of typically frugivorous species, with the European robin *Erithacus rubecula* being the most abundant (39.2%), while the blackbird *Turdus merula* and song thrush *T. philomelos* accounted for 4.8% and 2.7% of all recorded individuals, respectively. Over three seasons, 524 bird droppings were collected from standardized traps placed either beneath logs (log plots) or at randomly selected forest floor locations not associated with fleshy‐fruited plants (random plots). The survey showed that regardless of the season, the number of droppings differed significantly between plot types; they were significantly more abundant on log plots than on random plots (IRR = 0.26, 95% CI: 0.17–0.42, *p* < 0.001, Figure 2, Table 1). Across the entire study period, 73.7% of droppings (*n* = 386) were recorded beneath logs, compared to 26.3% (*n* = 138) on random plots (*χ* ^2^ = 10974.0, df = 1, *p* < 0.0001). Higher dropping abundance resulted in higher seed load; seed deposition was strongly spatially structured. Of all recovered seeds, 80.6% (*n* = 29, *χ* ^2^ = 806.4, df = 1, *p* < 0.0001) were found in log plots. Fleshy‐fruited plant species composition also differed between plot types, with limited overlap (Jaccard index = 0.375, Figure 3).

**TABLE 1:** Fixed effects from zero‐inflated negative binomial GLMM testing the effects of season and plot type on dropping counts (*N* = 1408). *p* < 0.05 in bold.

Fixed effect	Estimate	95% CI	*p*
Season 2022	1.02	0.71–1.46	0.914
Season 2023	1.50	1.06–2.11	**0.021**
Plot (random vs. log)	0.26	0.17–0.42	**< 0.001**
Season 2022 × plot	1.57	0.84–2.96	0.160
Season 2023 × plot	1.51	0.83–2.76	0.181

These results demonstrate that decaying logs that serve as perches influence the fine‐scale spatial distribution of bird droppings and the associated seed rain in temperate deciduous forests. Rotten logs, snags, and root plates after windthrows offer exposed structures attractive to forest‐dwelling birds as song‐posts during mating and breeding, as resting sites, or else as foraging platforms, since moist decaying logs support an abundance of arthropods, a potential food source (Czeszczewik and Walankiewicz 2016). Small frugivorous passerines are frequently associated with forests having many fallen logs and uprooted trees where they can perch (Urban and Smith 1989). As is well known, the amount of deadwood in managed forests is typically limited (Bobiec 2002; WISL 2022). Consequently, the spatial distribution of bird‐dispersed seeds is expected to differ from that observed in forests with a high abundance of down woody material.

Seed occurrence in bird droppings was low overall: only 4.6% (*n* = 24) of the droppings contained seeds (Figure 3). Despite its strong spatial aggregation around logs, the seed rain generated by birds was extremely low. For seed traps placed beneath logs, the seed rain was estimated at 0.63 seeds/m^2^/season, as compared with 0.15 seeds/m^2^/season for seed traps located on random plots. The overall seed rain for all seed traps pooled was 0.39 seeds/m^2^/season. This contrasts with values reported from secondary forests or forest edges showing a higher density of fleshy‐fruited plant seeds (2.64 seeds/m^2^/season, author's unpublished data). In comparison, pine marten *Martes martes* scats produced substantially higher seed rain (2.3 seeds/m^2^/season), with nearly half of the scats containing seeds (Kurek et al. 2026). Hence, the bird‐mediated seed input in closed‐canopy oak‐hornbeam forest appears to be less than that of other dispersal agents and in other habitats.

The low seed availability likely reflects environmental constraints characteristic of mature temperate deciduous forests, as fleshy‐fruited species require more light and grow better at forest margins and in canopy gaps (Eliáš 1984). As a result of light limitation in oak‐hornbeam forest, the distribution of fleshy‐fruited shrubs and trees is patchy, with low abundance and constancy (Thompson and Willson 1978). Low seed abundance and consequently low seed rain from frugivorous animals seem typical of forests characterized by near‐natural conditions (Jędrzejewska and Jędrzejewski 2001). Another reason may be that forest‐dwelling birds have relatively small territories (Tomiałojć 1993) and/or occur at low densities—“the habitat is not filled with birds” (Czeszczewik and Walankiewicz 2016), potentially restricting long‐distance seed transfer within continuous forest interiors. This interpretation is supported by the comparison with marten scats, as martens occupy much larger territories and may transport diaspores originating from adjacent habitats (Kurek et al. 2026).

Old decaying trees, snags and logs are recognized as key structural components supporting forest biodiversity (Stokland et al. 2012). They also are crucial elements shaping natural processes like seed dispersal. The presented results add a functional dimension: logs indirectly shape the spatial distribution of bird droppings and in turn the seed rain of fleshy‐fruited plants. Although the overall seed rain in mature deciduous forest is low, its spatial pattern is clearly nonrandom and linked to deadwood availability. This highlights a new aspect of woody debris in forest ecosystem functioning: three‐sided relations between birds, deadwood and fleshy‐fruited plants. Both findings—indirect interactions between birds and deadwood, and low seed rain generated by birds—add to our understanding of the role of birds in seed dispersal in temperate deciduous forests.

## Author Contributions

**Przemysław Kurek:** conceptualization (lead), data curation (lead), formal analysis (lead), funding acquisition (lead), investigation (lead), methodology (lead), software (lead), validation (lead), visualization (lead), writing – original draft (lead), writing – review and editing (lead).

## Funding

The study was supported by the National Science Centre (Poland, grant no. 2020/04/X/NZ8/00419).

## Conflicts of Interest

The author declares no conflicts of interest.

## Supporting information


**Appendix S1:** Example of seed trap located beneath log.

**Appendix S2:** Frequency of bird observations on logs used as perches, by species, based on camera‐trap photographs (*N* = 334).

**Data S1:** Raw data and R code used for the analyses, along with an attached README file.

## Data Availability

The datasets are available in Supporting Information.
