# Supplementary figures and images for: Decaying Logs Shape the Distribution of Bird‐Mediated Seed Rain in a Temperate Deciduous Forest
Source: Ecol Evol. 2026 Jul 29;16(8):e74087. doi: 10.1002/ece3.74087 (PMC13416749; doi:10.1002/ece3.74087)

Appendix 1. Example of seed trap located beneath log.


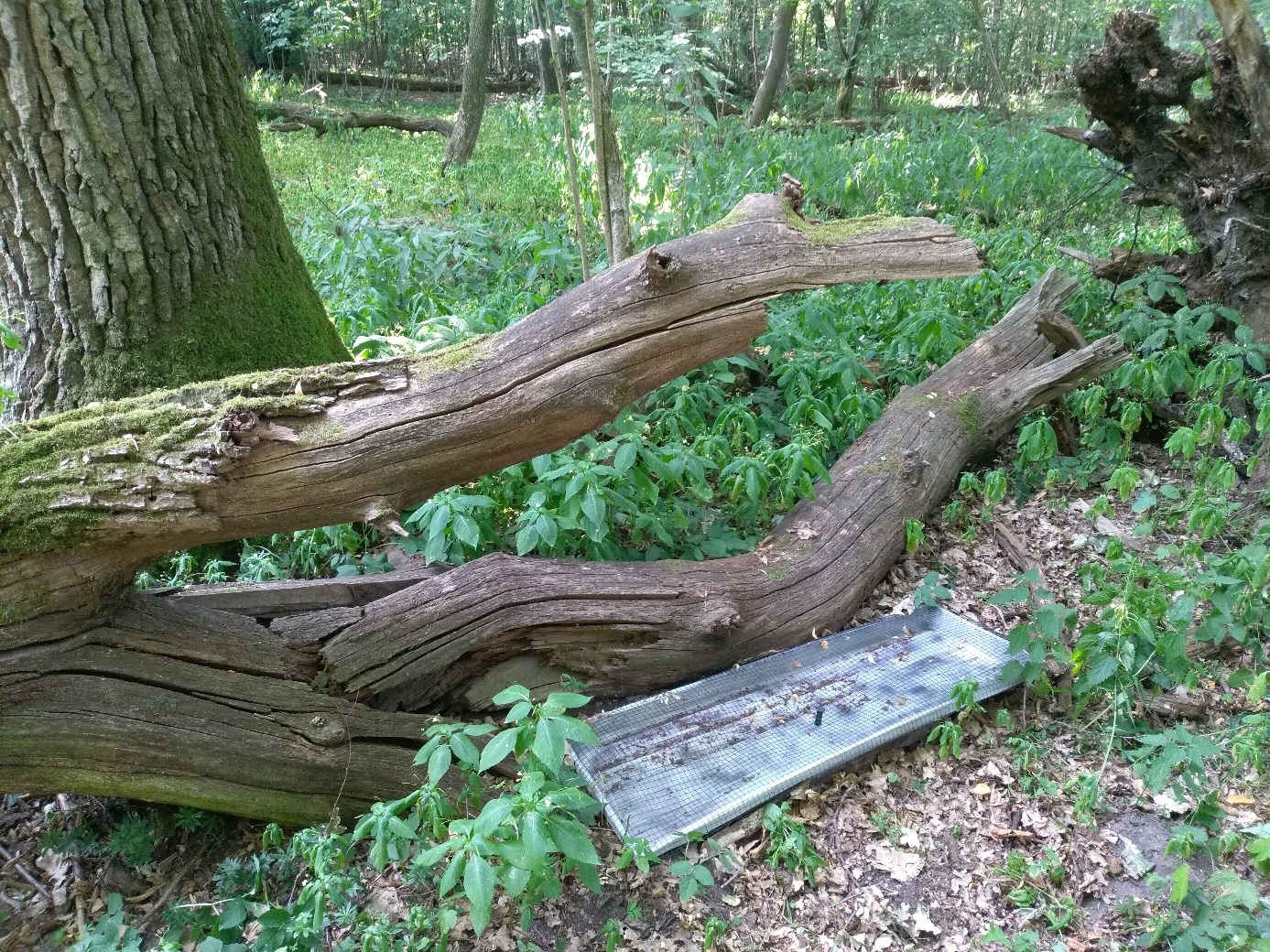

Supplement: Supplementary file 1 — Appendix S1: Example of seed trap located beneath log. [file ECE3-16-e74087-s003.docx]
