# Supplementary material for: Decaying Logs Shape the Distribution of Bird‐Mediated Seed Rain in a Temperate Deciduous Forest
Source: Ecol Evol. 2026 Jul 29;16(8):e74087. doi: 10.1002/ece3.74087 (PMC13416749; doi:10.1002/ece3.74087)
Supplement: Supplementary file 2 — Appendix S2: Frequency of bird observations on logs used as perches, by species, based on camera‐trap photographs (N = 334). [file ECE3-16-e74087-s001.docx]

Appendix 2. Frequency of bird observations on logs used as perches, by species, based on camera-trap photographs (N = 334).
