# Supplementary material for: Decaying Logs Shape the Distribution of Bird‐Mediated Seed Rain in a Temperate Deciduous Forest
Source: Ecol Evol. 2026 Jul 29;16(8):e74087. doi: 10.1002/ece3.74087 (PMC13416749; doi:10.1002/ece3.74087)
Supplement: Supplementary file 3 — Data S1: Raw data and R code used for the analyses, along with an attached README file. [file ECE3-16-e74087-s002.zip › R_codes.docx]

Main analyses

> library(glmmTMB)

> library(broom.mixed)

Variables:

> baza6$year<-as.factor(baza6$year)

> baza6$plot<-as.factor(baza6$plot)

> baza6$scat<-as.numeric(baza6$scat)

> if(od_ratio > 1.5){

+ cat("Overdispersion large, ->fit zero-inflated NB\n")

+ model <- glmmTMB(

+ scat ~ year * plot + (1 | tray),

+ ziformula = ~1,

+ family = nbinom2,

+ data = baza6

+ )

+ } else {

+ cat("Overdispersion acceptable, -> Poisson\n")

+ model <- model_pois

+ }

->Overdispersion large, -> fit zero-inflated NB

> summary(model)

Family: nbinom2 ( log )

Formula: scat ~ year * plot + (1 | tray)

Zero inflation: ~1

Data: baza6

AIC BIC logLik -2*log(L) df.resid

2130.6 2177.9 -1056.3 2112.6 1399

Random effects:

Conditional model:

Groups Name Variance Std.Dev.

tray (Intercept) 0.1335 0.3654

Number of obs: 1408, groups: tray, 32

Dispersion parameter for nbinom2 family (): 0.573

Conditional model:

Estimate Std. Error z value Pr(>|z|)

(Intercept) -0.82237 0.14258 -5.768 8.03e-09 ***

2022 0.01988 0.18348 0.108 0.9137

2023 0.40328 0.17501 2.304 **0.0212** *

plotref -1.33245 0.23418 -5.690 **1.27e-08** ***

2022:plotref 0.45357 0.32249 1.406 0.1596

2023:plotref 0.41115 0.30729 1.338 0.1809

---

Signif. codes: 0 ‘***’ 0.001 ‘**’ 0.01 ‘*’ 0.05 ‘.’ 0.1 ‘ ’ 1

Zero-inflation model:

Estimate Std. Error z value Pr(>|z|)

(Intercept) -17.8 3367.5 -0.005 0.996

>

> tidy(model, effects="fixed", conf.int=TRUE, exponentiate=TRUE)

# A tibble: 7 × 9

effect component term estimate std.error statistic p.value conf.low conf.high

*<chr>* *<chr>* *<chr>* *<dbl>* *<dbl>* *<dbl>* *<dbl>* *<dbl>* *<dbl>*

1 fixed cond (Intercept) 0.439 0.0626 -5.77 8.03e-9 0.332 0.581

2 fixed cond 2022 1.02 0.187 0.108 9.14e-1 0.712 1.46

3 fixed cond 2023 1.50 0.262 2.30 2.12e-2 1.06 2.11

4 fixed cond plotref 0.264 0.0618 -5.69 1.27e-8 0.167 0.418

5 fixed cond 2022:plotref 1.57 0.508 1.41 1.60e-1 0.837 2.96

6 fixed cond 2023:plotref 1.51 0.464 1.34 1.81e-1 0.826 2.76

7 fixed zi (Intercept) 0.0000000186 0.0000625 -0.00529 9.96e-1 0 Inf

Post-hoc tests

> library(emmeans)

> emm <- emmeans(model, ~ year)

> pairs(emm, adjust = "tukey")

contrast estimate SE df z.ratio p.value

2021 - 2022 -0.247 0.161 Inf -1.530 0.2770

2021 - 2023 -0.609 0.154 Inf -3.963 **0.0002**

2022 - 2023 -0.362 0.149 Inf -2.431 **0.0399**

> emm <- emmeans(model, ~ plot | year)

> pairs(emm, adjust = "tukey")

2021:

contrast estimate SE df z.ratio p.value

log - ref 1.332 0.234 Inf 5.690 <0.0001

2022:

contrast estimate SE df z.ratio p.value

log - ref 0.879 0.222 Inf 3.961 <0.0001

2023:

contrast estimate SE df z.ratio p.value

log - ref 0.921 0.199 Inf 4.622 <0.0001

Chi square test - goodness of fit

> chisq.test(x=c(29, 7), p=c(1, 35), rescale.p=T)

Chi-squared test for given probabilities

data: c(29, 7)

X-squared = 806.4, df = 1, p-value < **0.0001**

Figure 1:

p1<-ggplot(baza6, aes(x = year, y = scat, fill = plot)) +

geom_boxplot(outlier.shape = NA) +

geom_jitter(aes(color = plot),

+ width = 0.10,

+ alpha = 0.4,

+ size = 5

+ ) +

labs(

x = NULL,

y = "N of droppings") +

theme(legend.position = "none") +

theme_classic() +

theme(text = element_text(size = 18)) +

theme(axis.title.y = element_text(size = 16)) +

theme(axis.title.y = element_text(margin = margin(r = 10))) +

scale_y_continuous(breaks = scales::breaks_width(10))

# save in high resolution

ggsave("Fig.2.tiff", plot = p1, width = 10, height = 8, dpi = 300)
