# Supplementary material for: Decaying Logs Shape the Distribution of Bird‐Mediated Seed Rain in a Temperate Deciduous Forest
Source: Ecol Evol. 2026 Jul 29;16(8):e74087. doi: 10.1002/ece3.74087 (PMC13416749; doi:10.1002/ece3.74087)
Supplement: Supplementary file 3 — Data S1: Raw data and R code used for the analyses, along with an attached README file. [file ECE3-16-e74087-s002.zip › 00.readme.docx]

This readme file was generated on 2026-06-25 by PRZEMYSŁAW KUREK

# GENERAL INFORMATION

Title of Dataset: Number of droppings collected from seed traps

## Author/Principal Investigator Information

Name: Kurek Przemysław

ORCID: 0000-0002-5366-3057

Institution: Adam Mickiewicz University in Poznań, Poland

Address: Uniwersytetu Poznańskiego 6, 61-614 Poznań

Email: przkur1@amu.edu.pl

Date of data collection: 2021-2023

Geographic location of data collection:

52.803877°N, 17.141828°E, „Dębina” reserve near Wągrowiec, Poland.

Information about funding sources that supported the collection of the data:

National Science Centre (Poland, grant no. 2020/04/X/NZ8/00419).

# DATA & FILE OVERVIEW

## File List:

‘baza6’ – main dataset

‘R_codes’ – scripts description

# METHODOLOGICAL INFORMATION

## Description of methods used for collection/generation of data:

Bird droppings were collected in 2021–2023 during 22 surveys using 64 seed/droppings traps. Each trap consisted of a tray (40 × 120 cm, 0.48 m²) covered with wire mesh (mesh size 0.8 cm) to prevent post-dispersal seed removal. 32 seed traps were placed beneath downed woody material (DWM), including logs and thick branches with diameter > 10 cm at thinner end (log plots). The remaining 32 seed traps were placed at random forest-floor locations not associated with fleshy-fruited plants (random plots), to quantify the magnitude of bird-mediated seed rain independent of fruiting individuals. Traps were checked every 15–20 days from June to November, covering the fruiting phenology of fleshy-fruited herbs, shrubs and trees.

## Instrument- or software-specific information needed to interpret the data:

R software version 4.3.1 (R Core Team 2025)

glmmTMB

emmeans

# DATA-SPECIFIC INFORMATION FOR: baza6

Number of variables: 4

Number of cases/rows: 1408

Variable List:

-Year -> season: an explanatory categorical variable, season of the study 2021, 2022 and 2023

-Plot: an explanatory categorical variable, plots with seed traps beneath logs vs. random plots with seed traps out of the logs

-Scat: an dependent variable, number of bird droppings per seed trap per control

-Tray (in ms -> seed trap): random factor, id of seed trap

Specialized formats or other abbreviations used: .csv file
